# Real-world efficiency of lenvatinib plus PD-1 blockades in advanced hepatocellular carcinoma: an exploration for expanded indications

**DOI:** 10.1186/s12885-022-09405-7

**Published:** 2022-03-19

**Authors:** Xuqi Sun, Qi Zhang, Jie Mei, Ziliang Yang, Minshan Chen, Tingbo Liang

**Affiliations:** 1 Department of Hepatobiliary and Pancreatic Surgery, the First Affiliated Hospital, Zhejiang University School of Medicine, Hangzhou, 310006 China; 2 Zhejiang Provincial Key Laboratory of Pancreatic Disease, the First Affiliated Hospital, Zhejiang University School of Medicine, Hangzhou, China; 3 Department of Liver Surgery, Sun Yat-Sen University Cancer Center, Guangzhou, 510060 China

**Keywords:** Hepatocellular carcinoma, Lenvatinib, PD-1 blockades, Expanded indication, Prognosis

## Abstract

**Background:**

This study aimed to evaluate the efficiency and prognostic factors of lenvatinib plus programmed death 1 (PD-1) blockades in patients with advanced hepatocellular carcinoma (HCC), especially for those with tumor occupation ≥50% volume of liver (TO ≥50%) or invasion in Vp4, who were excluded from the trial KEYNOTE-524.

**Methods:**

We reviewed the clinical data of patients with unresectable HCC who received lenvatinib plus PD-1 blockades. The Kaplan-Meier method was performed to compare the progression-free survival (PFS) and the overall survival (OS). Cox proportional hazards model was adopted to identify independent prognostic factors.

**Results:**

The median PFS and OS of the enrolled 84 HCC patients (31 patients with TO ≥50% and 30 patients with Vp4 invasion) were 6.6 and 11.4 months respectively. TO ≥50% had significantly negative impact on the objective response rates (ORR) (*p* = 0.015). HCC patients with TO ≥50% had significantly worse PFS and OS than those with TO < 50% (both *p* value < 0.001). Conversely, invasion in Vp4 did not significantly affect the ORR, PFS or OS for HCC patients receiving lenvatinib plus PD-1 blockades (*p* = 0.419, 0.528 and 0.855). After multivariate analyses, TO ≥50% was the independent predictor for PFS and OS (both *p* value < 0.001). No significant correlation was found between any kind of AEs and TO ≥50% or invasion in Vp4.

**Conclusion:**

Lenvatinib plus PD-1 blockades can provide survival benefits for HCC patients with invasion in Vp4 and the indications of lenvatinib plus pembrolizumab may be further expanded. Locoregional treatments should be considered for patients with TO ≥50% during systemic therapy.

## Introduction

Hepatocellular carcinoma (HCC) ranks as the sixth most common malignancy worldwide, and is the third leading cause of cancer-related mortality [1]. HCC is extremely heterogeneous, the treatments of which mainly depend on tumor burden and liver function. For patients with early- or intermediate-stage HCC, several treatments can be accessed for curative intention including liver transplantation, hepatic resection and ablation [2]. Systemic therapy remains the main strategy for advanced HCC, which accounts for approximately 80% of HCC patients at diagnosis [2, 3]. In addition to sorafenib, the initial approved first-line drug for advanced HCC, lenvatinib was later approved as the first-line treatment for unresectable HCC due to its noninferior efficiency to sorafenib [4, 5].

In recent years, immunotherapies including programmed death 1 (PD-1) blockades have changed the landscape of treatments in advanced HCC [6]. The promising survival benefits from PD-1 blockades elicit clinical trials focusing on the combination of immunotherapies and targeted drugs such as atezolizumab plus bevacizumab, which was granted as prioritized first-line regimen for advanced HCC [6, 7]. Another combination therapy of lenvatinib plus pembrolizumab (KEYNOTE-524) also achieved the objective response rate (ORR) of 36.0% for unresectable HCC in phase Ib study, and its phase III trial LEAP-002 is currently underway [8]. To enroll patients most likely to benefit from the testing regimen, KEYNOTE-524 excluded patients with HCC having ≥50% liver occupation or Vp4 invasion [8]. In clinical practice, however, portal vein tumor thrombosis (PVTT) and high HCC burden exist in approximately 40% of patients at diagnosis due to the conceal onset of HCC [9, 10]. The prognosis of these patients remains poor, and therapeutic strategies are limited for them [9, 11]. For HCC patients with PVTT, systemic therapy is the only first-line therapy currently, but the expanding drugs provide limited data for HCC patients with Vp4 invasion or tumor occupation ≥50% volume of liver (TO ≥50%) [12].

In this study, we investigated the efficiency of lenvatinib plus PD-1 blockades in advanced HCC patients, especially for those with TO ≥50% or tumor thrombosis in Vp4. This research can complement the results of KEYNOTE-524 for patients with advanced HCC.

## Methods

### Patients

In this retrospective study, we reviewed HCC patients receiving lenvatinib plus PD-1 blockades in Sun Yat-sen University Cancer Center and the First Affiliated Hospital, Zhejiang University School of Medicine from January 2019 to June 2021. The inclusion criteria were as follows: 1) pathologically or clinically diagnosed as HCC according to the ESMO guidelines [2]; 2) with baseline imaging of HCC within 6 weeks before receiving lenvatinib plus PD-1 blockades and 3) with the Eastern Co-operative Oncology Group performance status score of 0–1. The patients were excluded if they had received immunotherapy before. Eventually, 84 HCC patients were included for further analysis. The demographic and clinical data were collected including age, gender, the status of hepatitis B surface antigen (HBsAg), Child Pugh class, tumor size, extrahepatic metastasis, serum alpha-fetoprotein (AFP) levels and adverse events (AEs). The invasion of Vp4 was defined as the thrombosis into the main trunk and/or the contralateral portal branch of the primary lesion [12]. Whether TO ≥50% was independently evaluated by two doctors. If their evaluation results were inconsistent, the third senior doctor should independently evaluate the tumor burden and make the final judgement. This study was approved by the Ethical Committee of Sun Yat-sen University Cancer Center and the First Affiliated Hospital, Zhejiang University School of Medicine.

### Systemic treatment and follow-up

All the patients received lenvatinib (12 mg/day for bodyweight ≥60 kg or 8 mg/day for bodyweight < 60 kg) plus PD-1 blockades. PD-1 blockades were intravenously administered at the standard dose as follows: pembrolizumab 200 mg (*n* = 10), toripalimab 240 mg (*n* = 37), sintilimab 200 mg (*n* = 23), nivolumab 100 mg (*n* = 13) or camrelizumab 200 mg (*n* = 1) every 3 weeks. Abdominal contrast enhanced computer tomography (CT) or magnetic resonance imaging (MRI) and chest enhanced CT were performed every 6–8 weeks during treatment. Tumor response was evaluated according to the Response Evaluation Criteria in Solid Tumors (RECIST 1.1) [13]. The ORR was the proportion of patients who confirmed complete response (CR) or partial response (PR). The AEs were assessed based on the Common Terminology Criteria for Adverse Events (CTCAE v5.0).

### Statistical analysis

The Kaplan-Meier method with log-rank test was performed to estimate the overall survival (OS) and progression-free survival (PFS). The OS was estimated from the date of initial combination therapy to the date of death or last follow-up. The PFS was calculated from the date of first dose of combination therapy to the date of progression or death. Chi-square test was used to compare baseline characteristics between subgroups classified by the presence of TO ≥50% or invasion in Vp4. Binary logistic regression analysis was used to evaluate the association between the ORR and tumor burden or invasion in Vp4. Univariate and multivariate Cox regression analyses were adopted to identify independent prognostic factors for OS and PFS. A two-tailed *P* value less than 0.05 was considered statistically significant. All statistical analyses were performed with the IBM SPSS, version 26.0 and the R software version 3.5.0.

## Results

### Baseline characteristics

The baseline characteristics of 84 patients are shown in Table 1. The median age was 53 years for the whole group. The patients were mainly male (*n* = 69). Most patients were infected with hepatitis B virus. Among the whole group, 85.7% (72/84) patients were Child Pugh A class, and the remaining were Child Pugh B class. 81.0% (68/84) patients were BCLC C stage and the others were BCLC B stage. In 42 patients with extrahepatic metastases, 29 patients had lung metastases, 14 patients had lymph node metastases, five patients had bone metastasis, three patients had peritoneal metastasis, one patient had renal metastasis and one patient had adrenal metastasis. In terms of treatments prior to lenvatinib plus PD-1 blockades, six patients received transarterial chemoembolization (TACE) plus sorafenib, three patients received sorafenib, one patient received apatinib and 27 patients received TACE. The shortest duration between lenvatinib plus PD-1 blockades and TACE was 1.17 months. In this cohort, 31 HCC patients had TO ≥50%, 30 HCC patients had tumor thrombosis in Vp4, and 12 patients had both TO ≥50% and tumor thrombosis in Vp4. Higher proportion of patients with TO ≥50% were Child Pugh B class compared with those with TO < 50% (*p* = 0.021). Patients with HCC invasion in Vp4 were younger than those without Vp4 invasion (*p* = 0.005). In addition, tumor thrombosis in Vp4 was negatively correlated with receiving prior systemic treatment (*p* = 0.012). Among 54 patients without Vp4 invasion, 37.0% (20/54) patients had Vp3 invasion, 7.4% (4/54) patients had Vp2 invasion and 55.6% (30/54) patients had no macrovascular invasion.

**Table 1:** Baseline characteristics of enrolled HCC patients

Variable	All patients	Tumor occupation ≥50%		Invasion with Vp4	
		Yes	No	Yes	No
Sample size	*n* = 84	*n* = 31	*n* = 53	*n* = 30	*n* = 54
Median age (range)	53 (25–78)	51 (29–78)	53 (25–72)	51 (29–78)	53 (25–78)
Sex					
Male	69 (82.1)	25 (80.6)	44 (83.0)	25 (83.3)	44 (81.5)
Female	15 (17.9)	6 (19.4)	9 (17.0)	5 (16.7)	10 (18.5)
HBsAg					
Positive	71 (84.5)	26 (83.9)	45 (84.9)	28 (93.3)	43 (79.6)
Negative	13 (15.5)	5 (16.1)	8 (15.1)	2 (6.7)	11 (20.4)
Child-Pugh class					
A	72 (85.7)	23 (74.2)	49 (92.5)	25 (83.3)	47 (87.0)
B	12 (14.3)	8 (25.8)	4 (7.5)	5 (16.7)	7 (13.0)
BCLC stage					
B	15 (17.9)	4 (12.9)	11 (20.8)	0 (0.0)	15 (27.8)
C	69 (82.1)	27 (87.1)	42 (79.2)	30 (100.0)	39 (72.2)
Extrahepatic metastasis					
Yes	42 (50.0)	13 (41.9)	29 (54.7)	12 (40.0)	30 (55.6)
No	42 (50.0)	18 (58.1)	24 (45.3)	18 (60.0)	24 (44.4)
AFP level ≥ 400 μg/ml					
Yes	45 (53.6)	20 (64.5)	25 (47.2)	19 (63.3)	26 (48.1)
No	39 (46.4)	11 (35.5)	28 (52.8)	11 (36.7)	28 (51.9)
Prior systemic treatment					
Yes	10 (11.9)	5 (16.1)	5 (9.4)	0 (0.0)	10 (18.5)
No	74 (88.1)	26 (83.9)	48 (90.6)	30 (100.0)	44 (81.5)
Prior TACE					
Yes	33 (39.3)	9 (29.0)	24 (45.3)	8 (26.7)	25 (46.3)
No	51 (60.7)	22 (71.0)	29 (54.7)	22 (73.3)	29 (53.7)
Maximum diameter of HCC ≥ 5 cm					
Yes	64 (76.2)	30 (96.8)	34 (64.2)	27 (90.0)	37 (68.5)
No	20 (23.8)	1 (3.2)	19 (35.8)	3 (10.0)	17 (31.5)

### Analysis of PFS and ORR

**Fig. 1:**
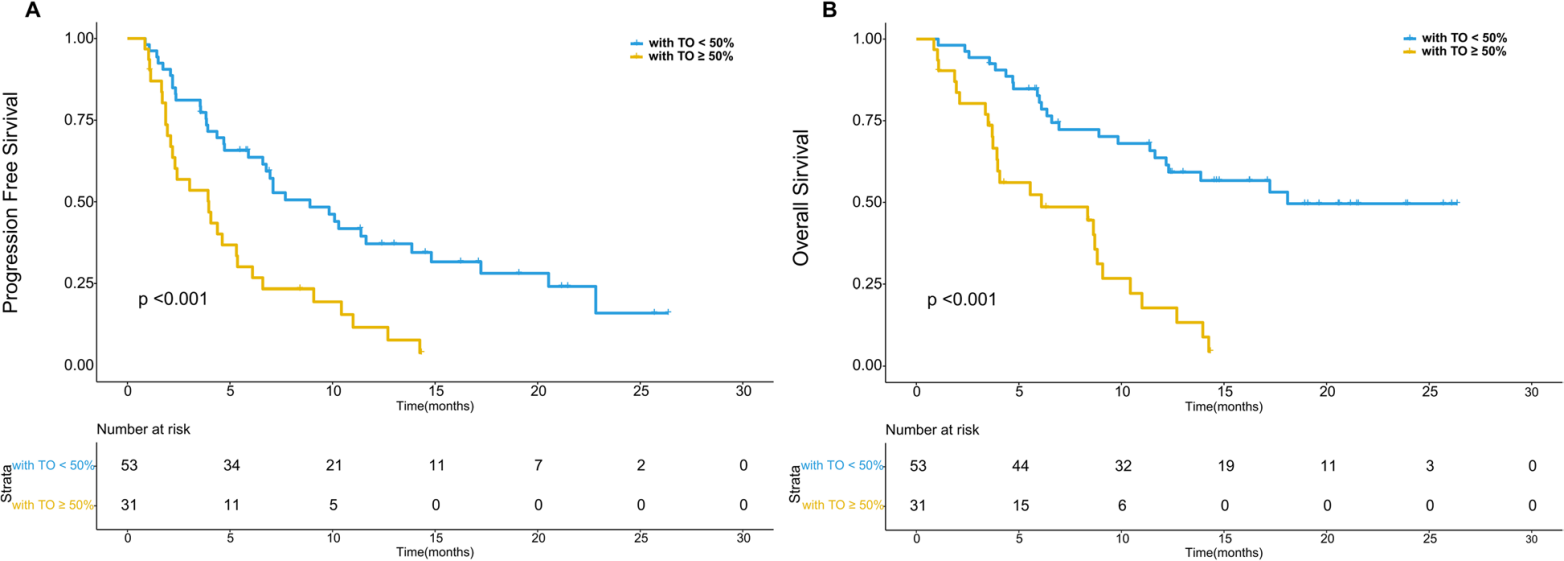
The survival curves of HCC patients with and without tumor occupation ≥50% volume of live (TO ≥50%). TO ≥50% had significantly negative impact on the PFS (**A**) and OS (**B**) (both *p* value < 0.001)

**Fig. 2:**
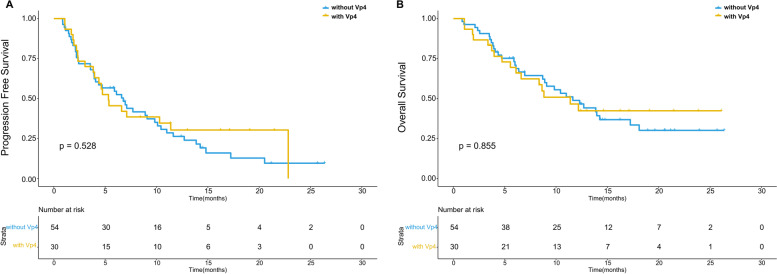
The survival curves of HCC patients with and without tumor thrombosis in Vp4. There was no significant difference between HCC patients with and without Vp4 invasion in terms of PFS (**A**) and OS (**B**) (*p* = 0.528 and 0.855)

The median follow-up time was 17.12 months (16.25 months for patients with Vp4 invasion, 18.93 months for patients without Vp4 invasion, 14.33 months for patients with TO ≥50% and 18.93 months for patients with TO < 50%). During follow-up, 76.2% (64/84) patients suffered from progression, and the median PFS was 6.6 months (95% confidence interval (CI), 4.3–8.9 months). The PFS was similar between patients receiving pembrolizumab and those receiving other PD-1 blockades (*p* = 0.328). The ORR was 20.2% (17/84) with one patient achieving CR, 16 patients achieving PR, 17 patients having stable disease, 17 patients having progressive disease and 33 patients without regular follow-up. HCC patients with TO ≥50% had significantly lower ORR than those with TO < 50% (*p* = 0.016). No significant difference was found between HCC patients with and without tumor thrombosis in Vp4 in terms of ORR (*p* = 0.278). We further compared the prognosis between patients classified by the presence of TO ≥50% or invasion in Vp4. The PFS of patients with TO ≥50% was significantly worse than that of patients with TO < 50% (*p* < 0.001) (Fig. 1A). Conversely, no significant difference was found between patients with and without Vp4 invasion (*p* = 0.528) (Fig. 2A). According to the results of multivariate analyses, only age and TO ≥50% had significant impact on the PFS of HCC patients receiving lenvatinib plus PD-1 blockades (*p* = 0.002 and < 0.001). Detailed data are listed in Table 2.

**Table 2:** Univariate and multivariate analyses of prognostic factors for PFS and OS

		PFS				OS			
		Univariate		Multivariate		Univariate		Multivariate	
Variables		HR (95%CI)	P	HR (95%CI)	P	HR (95%CI)	P	HR (95%CI)	P
Age, years	≥50	0.49 (0.30–0.80)	0.004	0.45 (0.27–0.76)	0.002	0.55 (0.31–0.97)	0.040	0.58 (0.33–1.04)	0.065
Sex	Female	1.57 (0.85–2.89)	0.152			0.94 (0.44–2.00)	0.863		
HBsAg	Positive	1.02 (0.51–2.08)	0.947			1.10 (0.47–2.60)	0.823		
Child Pugh class	B	1.19 (0.59–2.40)	0.636			1.83 (0.89–3.79)	0.103		
BCLC stage	C	0.76 (0.40–1.44)	0.397			1.40 (0.63–3.12)	0.413		
Extrahepatic metastasis	Yes	1.23 (0.75–2.01)	0.418			1.32 (0.74–2.33)	0.346		
AFP level ≥ 400μg/ml	Yes	1.02 (0.62–1.67)	0.941			1.01 (0.57–1.78)	0.981		
Prior systemic treatment	Yes	2.00 (0.98–4.06)	0.057			1.49 (0.70–3.19)	0.304		
Prior TACE	Yes	1.17 (0.71–1.93)	0.547			0.69 (0.38–1.25)	0.221		
Maximum diameter of ≥5 cm	Yes	1.45 (0.78–2.69)	0.236			1.86 (0.87–3.98)	0.110		
Tumor occupation ≥50%	Yes	2.50 (1.49–4.18)	< 0.001	2.66 (1.57–4.49)	< 0.001	3.98 (2.19–7.22)	< 0.001	3.90 (2.14–7.12)	< 0.001
Invasion with Vp4	Yes	0.85 (0.50–1.43)	0.528			0.95 (0.52–1.72)	0.855		

### Analysis of OS

The median OS was 11.4 months (95%CI, 7.9–14.9 months), and 57.1% (48/84) patients died during follow-up. No significant difference was found in OS between patients receiving pembrolizumab and those receiving other PD-1 blockades (*p* = 0.639). The median OS was 18.1 months for patients with TO < 50%, 6.1 months for patients with TO ≥50%, 11.63 months for patients without Vp4 invasion and 11.39 months for patients with Vp4 invasion. The 1-year survival rate was 60.7% for patients with TO < 50, 42.5% for patients with TO ≥50, 56.9% for patients without Vp4 invasion and 54.5% for patients with Vp4 invasion. As shown in survival curves, patients with TO ≥50% had significantly worse OS than those with TO < 50% (*p* < 0.001) (Fig. 1B). In terms of Vp4 invasion, there was no significant difference in OS between HCC patients with and without invasion in Vp4 (*p* = 0.855) (Fig. 2B). Although both age and TO ≥50% showed significant association with OS in univariate analyses (*p* = 0.040 and < 0.001), only TO ≥50% was the independent predictors for OS after multivariate analyses (*p* < 0.001). Detailed data are shown in Table 2.

### AEs

For patients with adequate following medical laboratory data, we assessed objective AEs of lenvatinib plus PD-1 blockades. The summary of AEs is presented in Table 3. In general, the most common AEs of any grade were bilirubin elevation (56.0%), elevated aspartate aminotransferase (55.1%) and proteinuria (52.6%). No significant correlation was found between any kind of AEs and TO ≥50% or Vp4 invasion.

**Table 3:** Summary of safety

	Percentages (%)
Elevated aspartate aminotransferase	
Any grade	55.1
Grade ≥ 3	18.4
Bilirubin elevation	
Any grade	56.0
Grade ≥ 3	12.0
Hypothyroidism	
Any grade	41.4
Grade ≥ 3	0
Anemia	
Any grade	26.4
Grade ≥ 3	3.8
Proteinuria	
Any grade	52.6
Grade ≥ 3	2.6

## Discussion

In this study, we compared the efficiency of lenvatinib plus PD-1 blockades between HCC patients with and without TO ≥50% or tumor thrombosis in Vp4, and we found that patients with TO ≥50% had significantly worse PFS or OS compared to those with TO < 50%. In addition, no significant difference was found between patients with and without invasion in Vp4 in terms of PFS and OS. To our knowledge, this was the first study to evaluate the utility of lenvatinib plus PD-1 blockades in HCC patients with TO ≥50% or tumor invasion in Vp4, who were excluded from the KEYNOTE-524, the phase Ib study of lenvatinib plus pembrolizumab.

With the conceal onset of HCC, most HCC patients are diagnosed at intermediate or advanced stage, and high tumor volume burden and PVTT are common in advanced HCC [9, 14]. The prognosis of HCC patients with PVTT remains dismal, whose OS is only 2–4 months with best supportive care [15, 16]. Although oncologists have explored the utility of hepatectomy and locoregional treatments in HCC patients with tumor thrombosis in Vp4, no survival benefits are observed for hepatectomy and high risk of hepatic failure limits the application of TACE in HCC patients with invasion in the main portal trunk or the first-order branches [17, 18]. According to current clinical guidelines, systemic therapy is recommended as the first-line treatment for HCC patients with PVTT [2]. The restrictions for sorafenib do not include portal vein invasion, however, lenvatinib is not recommended for patients with main portal vein invasion [2]. Kuzuya et al. have reported that lenvatinib can achieve better efficiency than sorafenib for HCC patients with Vp3/4 invasion, which suggested the application value of lenvatinib in HCC with Vp3/4 invasion [19]. Although Choi et al. found that hepatic arterial infusion chemotherapy (HAIC) can achieve better ORR and OS than sorafenib in HCC patients with PVTT, few studies have evaluated the efficiency of lenvatinib plus PD-1 blockades for HCC patients with tumor thrombosis in Vp4 [20].

Lenvatinib, an oral multikinase inhibitor, targets multiple molecules such as vascular endothelial growth factor receptor 1–3 and fibroblast growth factor [8, 21]. PD-1 blockades have revolutionized the treatments of advanced HCC in recent years, which can reactive the suppressed immune microenvironment [6]. Lenvatinib and PD-1 blockades have synergistic anti-tumor effects. Lenvatinib can decrease immunosuppressive impacts of HCC, which in turn improve the anti-tumor effects of PD-1 blockades [22]. The results of phase Ib study of lenvatinib plus pembrolizumab showed promising survival benefits for unresectable HCC, and subgroup analyses indicated that the ORR was consistent between patients with and without macroscopic portal vein invasion (37.5% vs 35.7%) [8]. To enroll patients most likely to benefit from this testing regimen, HCC patients with TO ≥50% or Vp4 invasion were excluded from this trial, however, these patients account for a majority of advanced HCC patients. Previous studies have found the OS of HCC patients with TO ≥50% or Vp4 invasion was worse than that of HCC patients without TO ≥50% or Vp4 invasion when receiving lenvatinib alone [12, 23, 24]. As for PFS, there is no consistent conclusion.

Although previous researches have evaluated potential of lenvatinib for the expanded indication, these studies did not further assess the impact of TO ≥50% and Vp4 invasion respectively. In current study, we found that there was no significant difference between HCC patients with and without Vp4 invasion in terms of ORR, PFS and OS when receiving lenvatinib plus PD-1 blockades. The ORR was 33.3% in this study, which is similar with the ORR reported by Huang et al. [25]. In addition, they also evaluated organ-specific response rates (OSRR) for patients with unresectable HCC receiving first-line lenvatinib plus PD-1 antibodies, and the OSRR of macrovascular tumor thrombi could reach to 54.5%, which was higher than that of lung metastases (37.5%), intrahepatic tumors (32.8%) and lymph node metastases (33.3%) [25]. These results indicate that tumor thrombosis in portal vein has better sensitivity to the combination therapy compared with HCCs in other sites, which can explain why the Vp4 invasion did not significantly affect the efficiency of lenvatinib plus PD-1 blockades in this study. The median OS and 1-year survival rate in our study was worse than those in the trial KEYNOTE-524 which may be due to the heavier tumor burden and relatively poor liver function of enrolled patients [8].

As for tumor burden, we found HCC patients with TO ≥50% had significantly worse ORR, PFS and OS compared with those with TO < 50%. These results indicate that lenvatinib plus PD-1 blockades may not be the optimal treatment for HCC patients with TO ≥50%. Similarly, Huang et al. have found that the ORR of combined lenvatinib with PD-1 antibody is worse in intrahepatic lesions than vascular invasion or extrahepatic metastases [25]. Another study also found that intrahepatic tumors are less responsive to immune checkpoint inhibitors than extrahepatic metastases in HCC patients [26]. The anti-tumor ability of PD-1 blockades mainly depends on reactivating exhausted T cells, and the immune microenvironments of different organs can affect the therapeutic effects of PD-1 blockades [26]. Due to the special physiological function, the liver is consistently exposed to new antigens, which creates the tolerogenic microenvironment of the liver [27]. Studies have also found liver metastases have lower response rates to anti-PD-1 immunotherapies than primary or other metastatic lesions in other solid tumors such as melanoma and non-small cell lung cancer [28]. Besides, high tumor volume might limit locoregional concentration of drugs, which could compromise the efficiency of lenvatinib and PD-1 blockades. Therefore, systemic therapy alone may be not sufficient for large HCC, and locoregional treatments such as TACE or HAIC should be considered simultaneously during systemic treatments.

There are several limitations for this study. First, it was a retrospective study, which may lead to unintentional biases and lack of some information for the evaluation of AEs. To avoid subjective recall biases, we only evaluated the laboratory AEs for patients with laboratory follow-up records, which caused the higher rates of AEs in this study since the follow-up laboratory tests were given based on the considerations of physicians. Second, PD-1 blockades were from different manufacturers, but patients receiving pembrolizumab had similar prognosis with those receiving other PD-1 blockades. The results therefore should be genuine. Third, the ORR of lenvatinib plus PD-1 blockades in this study was lower than those reported in previous studies, which might be due to 33 patients without regular follow-up for evaluating efficiency [8, 25]. Among 51 patients receiving regular follow-up, the response rate was 33.3% (17/51). Forth, the sample size was limited for this research, and most patients were infected with HBV. Further multicenter study with sufficient sample size is expected to evaluate whether these results can be applied to HCC patients with different etiologies.

In conclusion, lenvatinib plus PD-1 blockades can provide survival benefits for HCC patients with tumor thrombosis in Vp4, which indicates the indications of lenvatinib plus pembrolizumab may be further expanded. On the contrary, this combination regimen may not be sufficient enough for HCC patients with TO ≥50%, and other locoregional treatments should be considered simultaneously during systemic treatments.

## Data Availability

The datasets used during the current study are available from the corresponding author on reasonable request.

## Abbreviations

HCC: Hepatocellular carcinoma
PD-1: Programmed death 1
ORR: Objective response rate
PVTT: Portal vein tumor thrombosis
TO ≥50%: Tumor occupation ≥50%
HBsAg: Hepatitis B surface antigen
AFP: Alpha-fetoprotein
AEs: Adverse events
CT: Computer tomography
MRI: Magnetic resonance imaging
RECIST 1.1: Response Evaluation Criteria in Solid Tumors 1.1
CR: Complete response
PR: Partial response
CTCAE v5.0: Common Terminology Criteria for Adverse Events version 5.0
OS: Overall survival
PFS: Progression-free survival
CI: Confidence interval
TACE: Transarterial chemoembolization
HAIC: Hepatic arterial infusion chemotherapy
OSRR: Organ-specific response rates
